# Prognostic Value of *Isocitrate Dehydrogenase* Mutations in Myelodysplastic Syndromes: A Retrospective Cohort Study and Meta-Analysis

**DOI:** 10.1371/journal.pone.0100206

**Published:** 2014-06-17

**Authors:** Jie Jin, Chao Hu, Mengxia Yu, Feifei Chen, Li Ye, Xiufeng Yin, Zhengping Zhuang, Hongyan Tong

**Affiliations:** 1 Department of Hematology, the First Affiliated Hospital of Zhejiang University, Hangzhou, People’s Republic of China; 2 Institute of Hematology, Zhejiang University School of Medicine, Hangzhou, People’s Republic of China; 3 Surgical Neurology Branch, National Institute of Neurological Disorders and Stroke, National Institutes of Health, Bethesda, Maryland, United States of America; UT MD Anderson Cancer Center, United States of America

## Abstract

**Background:**

Recent genomic sequencing efforts have identified a number of recurrent mutations in myelodysplastic syndromes (MDS) that may contribute to disease progression and overall survival, including mutations in *isocitrate dehydrogenases 1* and *2* (*IDH1* and *IDH2*).

**Methods:**

Pretreatment bone marrow (BM) samples were acquired from mononuclear cells in 146 adult patients with de novo MDS from January 2006 to June 2013. Polymerase chain reaction (PCR) and direct sequencing were performed on exon 4 of *IDH1/2* genes and mutation status was correlated with overall survival (OS) and leukemia-free survival (LFS). We then performed a meta-analysis combining previously published and current studies to explore the effect of *IDH* mutations on OS and LFS in MDS.

**Results:**

In our study, somatic mutations of either *IDH* gene were discovered in 11 MDS patients (7.53%) and were significantly correlated with poorer OS (*P* = 0.007). *IDH* mutations were specifically associated with a poorer OS in the intermediate-1 risk group by the International Prognostic Scoring System (IPSS) (*P* = 0.039). In addition, we discovered decitabine achieved a better therapeutic effect compared to other treatments in *IDH* mutation-positive patients (*P* = 0.023). We identified six previous studies of *IDH* mutations in MDS. A meta-analysis of these studies included 111 MDS patients *IDH* mutations and 1671 MDS patients with wild-type *IDH1/2*. The hazard ratios (HRs) of OS and LFS for patients with *IDH* mutations were 1.62 (95% CI, 1.27–2.09) and 2.21 (95% CI, 1.48–3.30), respectively.

**Conclusion:**

The results from our study and the meta-analysis provide firm evidence that *IDH* mutations are significantly associated with poorer clinical outcomes in MDS. Identification of *IDH* mutations may be pivotal for better risk stratification in MDS patients and improving IPSS score. Additionally, hypomethylating agents may be an effective treatment option for MDS patients with *IDH* mutations.

## Introduction

Myelodysplastic syndromes (MDS) comprise a heterogeneous group of hematological disorders defined by blood cytopenias due to ineffective hematopoiesis and an increased risk of developing acute myeloid leukemia (AML) [1], [2]. Despite recent advances in therapeutic methods, treatments for MDS are currently tailored to individual patient needs, making the precise forecast of the prognosis an important component of treating patients [3]. Current prognostic scoring systems for patients with MDS are mainly based on karyotypic abnormalities and certain clinical features that are used to stratify risk. Although existing systems such as the IPSS [4], Revised-IPSS [5] and WHO-classification-based Prognostic Scoring System (WPSS) [6] help to estimate patient outcomes and guide treatment decisions, there remains significant variability in prognosis. Hence, novel molecular markers may offer more precise cancer phenotypes and more accurate estimation of prognosis for MDS patients.

Until now, the pathogenesis of MDS has not been clearly identified, but it is generally acknowledged that genetic mutations and dysfunction of gene contribute to the development and progression of this preleukemic disease [7], [8]. Genetic mutations are not currently used in estimating prognosis in MDS but are likely key determinants of overall survival and clinical phenotypes [9]. Therefore, contributing gene mutations may supplement current prognostic systems to improve the prediction of prognosis for MDS patients.

IDH 1/2 are key metabolic enzymes that convert isocitrate to α-ketoglutarate (α-KG or 2-oxoglutarate, 2-OG), which is an essential cofactor for α-KG dependent dioxygenases [10], [11]. These enzymes are associated with diverse cellular processes such as adapting to histone deacetylation, hypoxia, and DNA demethylation [12]. Therefore, *IDH* mutations may be causally linked to the clinical impacts of patients with MDS. We identified 146 patients with primary MDS and analysed *IDH* mutation status with OS and LFS. We then performed a meta-analysis combining our data with those of the published literature to furnish a more accurate estimation of the relationship between *IDH* mutations and MDS.

## Methods

### Patients

One hundred and forty-six adult patients with de novo MDS diagnosed according to World Health Organization (WHO) 2001 criteria [13] were recruited at the department of hematology, the First Affiliated Hospital of Zhejiang University. MDS patients were stratified by cytogenetic risk according to IPSS protocols [4]. All of the subjects were well-informed about the study and provided written informed consent to participate this study. This study was approved by the Institutional Review boards of the First Affiliated Hospital of Zhejiang University. Follow-up data were obtained by telephoning and reviewing patients’ medical records. 7 of 146 patients (4.79%) were lost to follow-up. Treatments were performed for patients including chemotherapy regimens (the GAA regimen (granulocyte-colony stimulating factor (G-CSF) 200 µg/m^2^ per day on days 1–14, aclacinomycin 10 mg per day on days 1–14; cytarabine 10 mg/m^2^, days 1–14; n = 2); the GHA regimen (G-CSF 200 µg/m^2^ per day on days 1–14, homoharringtonine 1 mg/m^2^ per day on days 1–14, cytarabine 10 mg/m^2^ per day on days 1–14; n = 6); the DA or IA regimen (daunorubicin 40–45 mg/m^2^ per day on days 1–3 or idarubicin 8–12 mg/m^2^ per day on days 1–3, cytarabine 100 mg/m^2^ per day on days 1–7; n = 8); decitabine (20 mg/m^2^/day, days 1–5 or 15 mg/m^2^, q8 h, days 1–3; n = 44)) and supportive care (antibiotics, androgen, all-*trans* retinoic acid, blood product transfusion and iron chelation therapy; n = 86).

### Mutational Analyses for the IDH1 and IDH2 Genes

Pretreatment BM specimens were enriched for mononuclear cells using Ficoll density gradient centrifugation. Genomic DNA was extracted from cryopreserved mononuclear cells using the DNA Kit (Sangon, Shanghai, China) according to the manufacturer’s instructions. Approximately 100 ng of DNA was used for each PCR reaction. The primer pairs were the same as those designed by Patnaik et al [14]. The PCR amplification conditions were as follows: 95°C for 5 minutes; followed by 40 cycles of 95°C for 30 seconds, 60°C for 30 seconds, and 72°C for 30 seconds; and finally, 72°C for 5 minutes. PCR products were directly sequenced on both strands using an ABI 3730 automatic sequencer by Sangon.

### Statistical Analysis

OS end-points were defined as the time from diagnosis of MDS to death due to any cause or to the time of last follow-up. LFS end-points were defined as the time from MDS diagnosis to either AML progression or death or failure or alive without disease progression at the date of most recent follow-up. Length of survival comparisons were analyzed using the Kaplan-Meier method. For categorical parameters, overall group differences were compared with the χ^2^ or Fisher exact test. For continuous variables, overall group differences were evaluated with the Mann-Whitney *U* test. A Cox proportional hazards model was performed to evaluate the effect of endpoint on OS and LFS for multivariate analysis. Statistical analysis was performed with SPSS 16.0 software package (SPSS, Chicago, USA). All tests were 2-tailed, and a *P*-value of less than 0.05 was considered statistically significant.

### Meta-analysis of *IDH1/2* Mutations in MDS

**Figure 1:**
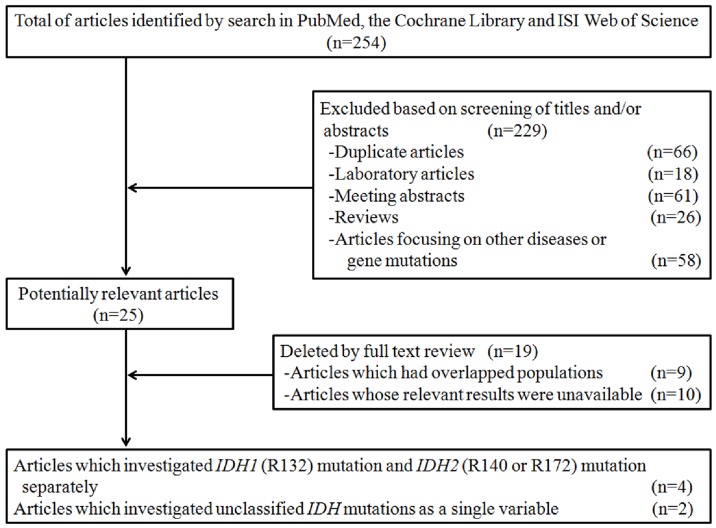
Flow diagram of study selection.

To further assess the relationship between *IDH1/2* mutations and MDS risk, we conducted a meta-analysis combining our study data with published studies on *IDH* mutations in MDS [3], [14], [15], [16], [17], [18]. Two independent reviewers (CH and MXY) performed a systematic literature search using ISI Web of Science, PubMed and the Cochrane Library for relevant papers published before December 2013 by the search term “(MDS OR myelodysplastic syndrome OR preleukemia OR myelodysplasia) AND (*IDH1* OR *IDH2*).” Reviews and references of related articles were checked for missing information. Eligible papers met all the following criteria: (1) assessed the association between *IDH1/2* mutations and outcomes in MDS; (2) detailed survival information of patients with *IDH1* or *IDH2* mutations; (3) reported the study in English. Animal studies, letters to the editor without original data, reviews and case reports were excluded. In the event of multiple publications from overlapping study populations or the same study, only the one with the largest sample size was selected (Figure 1).

The following data were extracted from each article: first author’s name, year of publication, country of origin, participant gender, participant age, sample size, MDS subtype, criteria for classification of MDS, karyotypes and IPSS classification. If the required data for the meta-analysis were not available in the published study, we contacted the corresponding authors for missing data.

A general variance-based method and a mathematical HR approximation method [19] in this meta-analysis were simultaneously used to estimate the summary HRs and their 95% CIs for the combined large sample set. Assessing heterogeneity and choosing fixed-effect or random-effect were performed as described previously [20]. Sensitivity analysis was conducted by sequential omission of individual studies and evaluated influence of each study on the stability of the results. Cumulative analysis was performed by assortment of publication time. Publication bias was assessed by funnel plot and Egger’s test [21], [22]. All statistical analyses were carried out in STATA 11.0 statistical software (Stata Corporation, College Station, Texas), and a *P*-value less than 0.05 was considered significant.

## Results

### Patient Characteristics

The current study included 146 patients (85 men and 61 women). The median age was 55 years (range 18–85). According to the WHO criteria, 7 (4.79%) patients were classified as refractory anemia (RA), 3 (2.05%) as RA with ringed sideroblasts (RARS), 50 (34.25%) as refractory cytopenia with multilineage dysplasia (RCMD), 44 (30.14%) as RA with excess blasts type 1 (RAEB1) and 42 (28.77%) as RAEB2 [23]. Cytogenetic results were available for 141 patients. The data demonstrated a low risk in 99 patients, an intermediate risk in 26 patients and a high risk in 16 patients. IPSS risk distributions were: low risk in 7 patients (4.97%), intermediate-1 risk in 76 patients (53.90%), intermediate-2 risk in 46 patients (32.62%) and high risk in 12 patients (8.51%).

### *IDH1/2* Mutations in MDS and Association with Clinical Outcomes

*IDH1/2* mutations were identified in eleven (7.53%) MDS patients, six (4.11%) had mutations in *IDH1* and five (3.42%) had mutations in *IDH2* (Table 1). Among MDS patients with *IDH1/2* mutations, two (18.18%) were classified as RAEB1, seven (63.64%) as RAEB2 and two (18.18%) as RCMD. Seven MDS patients (64.64%) with *IDH* mutations had a normal karyotype. Of the four patients with *IDH1/2* mutations and abnormal karyotypes, three (75%) carried a −7/7q-. All MDS patients with *IDH1* mutations carried an *IDH1* R132C mutation, whereas all patients with *IDH2* mutations carried an *IDH2* R140Q mutation. *IDH1/2* mutants carried significantly more bone marrow (BM) blasts than MDS patients with wild-type *IDH1/2* (*P* = 0.022); no significant differences were observed in age, sex, white blood cell (WBC) count, hemoglobin, platelet count, WHO subtype, cytogenetics or IPSS.

**Table 1:** Characteristics of patients with MDS.

	*IDH1* mutation	*IDH2* mutation	Wild-type	*P*
	(n = 6)	(n = 5)	(n = 135)	
Sex				0.360
Male	4	4	77	
Female	2	1	58	
Median age, years (range)	69(46–74)	61(36–78)	55(18–85)	0.122
Median WBC, ×10^9^/L (range)	4.1(2.2–15.6)	3(1.3–5.7)	2.8(0.4–26.4)	0.221
Median hemoglobin, g/L (range)	94(60–102)	74(60–82)	80(39–169)	0.850
Median platelets, ×10^9^/L (range)	56(20–86)	89(27–484)	70(4–542)	0.891
Median blasts, %(range)	11.8(5–18)	13(2–19.5)	6(0.5–18.5)	0.022
WHO subtype				0.121
RA	0	0	7	
RARS	0	0	3	
RCMD	0	2	48	
RAEB1	2	0	42	
RAEB2	4	3	35	
Karyotype classification				0.087
Low risk	4	4	91	
Intermediate risk	0	0	26	
High risk	2	1	13	
IPSS				0.364
Low risk	0	0	7	
Intermediate 1	2	2	72	
Intermediate 2	2	3	41	
High risk	2	0	10	

Abbreviations: MDS, myelodysplastic syndromes; WHO, World Health Organization; RA, refractory anemia; RARS, RA with ringed sideroblasts; RCMD, refractory cytopenia with multilineage dysplasia; RAEB-1, RA with excess blasts type 1; WBC, white blood cell count; IPSS, International Prognostic Scoring System.

**Figure 2:**
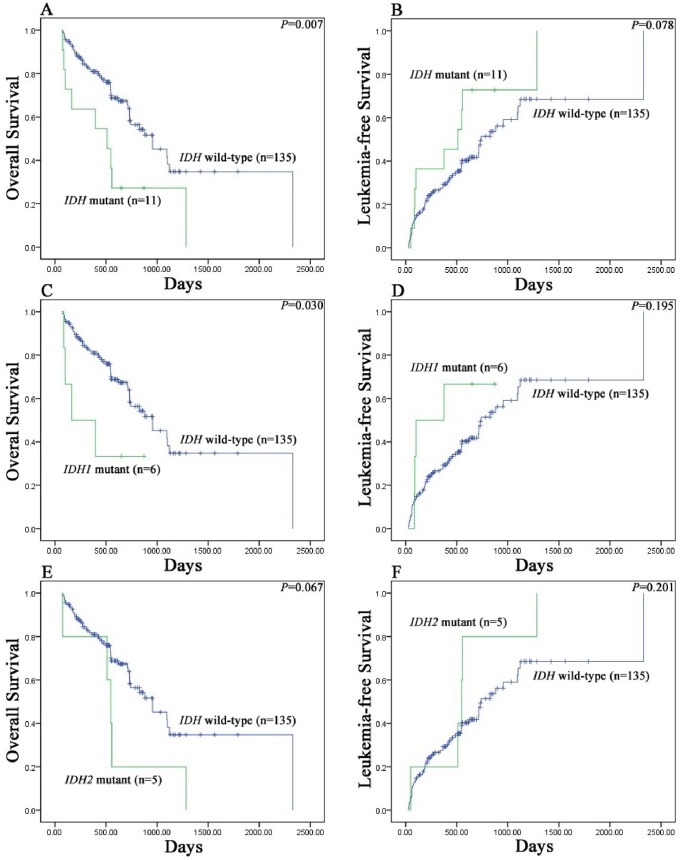
Kaplan–Meier survival curves for survival of MDS patients. (A) Overall survival data for MDS patients stratified by *IDH1/2* mutational status. (B) Leukemia-free survival data for MDS patients stratified by *IDH1/2* mutational status. (C) Overall survival data for MDS patients stratified by *IDH1* mutational status. (D) Leukemia-free survival data for MDS patients stratified by *IDH1* mutational status. (E) Overall survival data for MDS patients stratified by *IDH2* mutational status. (F) Leukemia-free survival data for MDS patients stratified by *IDH2* mutational status.

**Figure 3:**
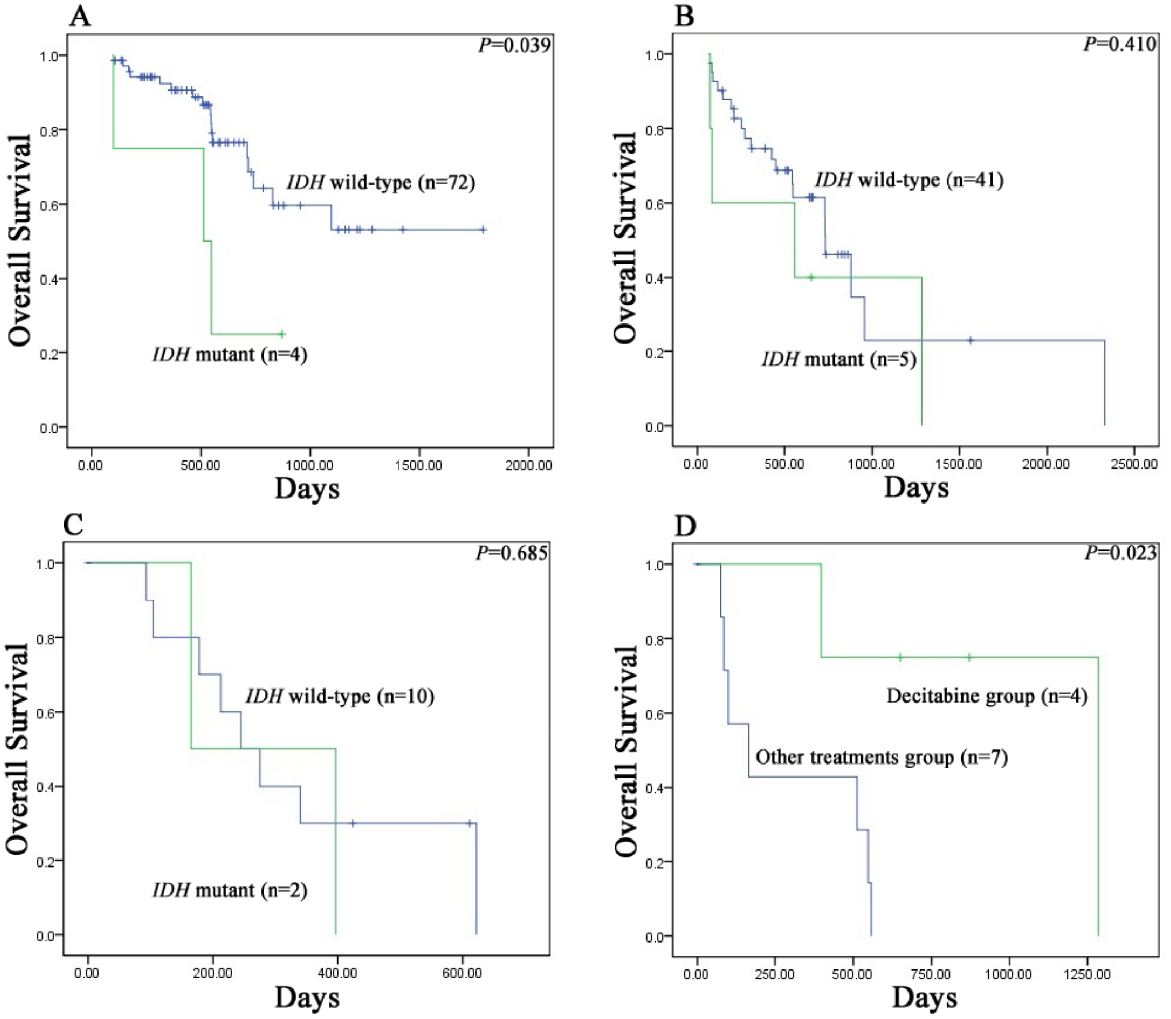
Kaplan–Meier survival curves for overall survival of MDS patients. (A) Overall survival of MDS patients in the intermediate-1 risk group of IPSS. (B) Overall survival of MDS patients in the intermediate-2 risk group of IPSS. (C) Overall survival of MDS patients in the high risk group of IPSS. (D) Kaplan–Meier survival of *IDH* mutant patients with decitabine chemotherapy compared with other treatments.

The median survival time was 512 days (range 100–924 days) in the *IDH1/2* mutant group and 956 days (range, 632–1280 days) in the wild-type *IDH1/2* group. Survival analysis demonstrated MDS patients harboring *IDH1/2* mutations had significantly shorter OS compared to patients with wild-type *IDH1/2* (*P* = 0.007) (Figure 2A). Further, we found *IDH1* mutations negatively affected OS in MDS (*P* = 0.030) rather than *IDH2* mutations (*P* = 0.067) (Figure 2C, E). The presence of *IDH1/2* mutations did not influence the LFS (*P* = 0.078, 0.195 and 0.201, respectively) (Figure 2B, D, F). Interestingly, our data showed the presence of *IDH1/2* mutations was an adverse predictor of OS in the intermediate-1 risk group of IPSS (*P* = 0.039) (Figure 3A), but not in the intermediate-2 risk (*P* = 0.410) (Figure 3B) or high risk (*P* = 0.685) (Figure 3C) group. Our results also indicated that decitabine achieved a better therapeutic effect in *IDH1/2* mutation-positive patients compared to other treatments (including: GHA regimen, n = 3; GAA regimen, n = 2; supportive care, n = 2) (*P* = 0.023) (Figure 3D).

Multivariable analysis including *IDH* mutations, age, WBC count, hemoglobin, platelet count, BM blast count, cytogenetic changes and IPSS class showed HRs of *IDH1/2* mutations for OS and LFS were 1.83 (95%CI 0.86–3.92) (*P* = 0.118) and 1.18 (95%CI 0.56–2.50) (*P* = 0.662), respectively. In addition, HRs of mutant *IDH1* for OS and LFS were 1.62 (95%CI 0.55–4.81) (*P* = 0.383) and 1.07 (95%CI 0.37–3.09) (*P* = 0.903), and HRs of mutant *IDH2* for OS and LFS were 1.93 (95%CI 0.70–5.35) (*P* = 0.206) and 1.23 (95%CI 0.44–3.40) (*P* = 0.692), respectively.

### Meta-analysis Results

As shown in Figure 1, six studies and our data covering a total of 1782 subjects (111 with *IDH1/2* mutations, 1671 with wild-type *IDH*) were included in the meta-analysis. Two of them were from United States [3], [14], one from Germany [15] and three from Asia [16], [17], [18] (Table 2). Two of these studies found a correlation between *IDH1/2* mutations and adverse prognosis in MDS [14], [15]. For all studies in this meta-analysis, MDS were diagnosed by the WHO [13] or FAB (French-American-British) criteria [24].

**Table 2:** Main characteristics of studies involving in the meta-analysis.

			age	Sex	MDS subtype	MDS classification	IPSS karyotype	IPSS	Number of *IDH* mutation
study	Country	number	(range)	female	male	RA/RARS	RCMD	RAEB/RAEB-t	Others		Good/Intermediate/	Low+Int1/Int2+	(*IDH1/IDH2*)
											Poor/Unknown	High/Unknown	
Thol	Germany	193	NR(36–92)	119	74	38/20	30	53/0	52	WHO	109/20/23/41	96/51/46	7(7/0)
(2010) [15]													
Bejar	United	439	70(NR)	133	306	197/47	0	160/34	1	FAB	310/55/67/7	295/133/11	15(6/9)
(2011) [3]	States												
Lin	China	82	NR (20–85)	35	47	8	35	34/0	5	WHO	62/11/7/2	59/21/2	5(2/3)
(2012) [16]													
Patnaik	United	277	71 (21–91)	78	199	0/56	130	77/0	14	WHO	NR	190/87/0	34(8/26)
(2012) [14]	States												
Lin	China and	168	NR(60–75)	55	113	NR	38	119/0	NR	WHO	NR	73/77/18	17(7/10)
(2013) [17]	Japan												
Lin	China	477	66(18–98)	158	319	207	0	161/56	53	FAB	274/87/85/1	256/190/1	22(3/19)
(2013) [18]													

Abbreviations: MDS, myelodysplastic syndromes; WHO, World Health Organization; FAB, French American British classification; RA, refractory anemia; RARS, RA with ringed sideroblasts; RCMD, refractory cytopenia with multilineage dysplasia; RAEB, RA with excess blasts; RAEB-t, RAEB in transformation; IPSS, International Prognostic Scoring System; *IDH*, *isocitrate dehydrogenase*.

**Figure 4:**
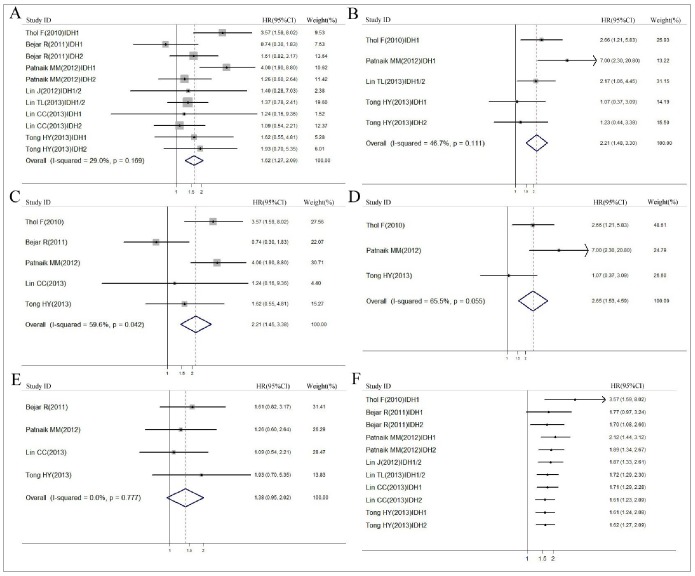
Forest plots describing the association between *IDH* mutations and MDS. (A) Forest plots of HR and 95% CI for *IDH1/2* mutations in MDS comparing with *IDH* wild-type by OS endpoints. (B) Forest plots of HR and 95% CI for *IDH1/2* mutations in MDS comparing with IDH wild-type by LFS endpoints. (C) Forest plots of HR and 95% CI for *IDH*1 mutations in MDS comparing with *IDH*1 wild-type by OS endpoints. (D) Forest plots of HR and 95% CI for *IDH*1 mutations in MDS comparing with *IDH*1 wild-type by LFS endpoints. (E) Forest plots of HR and 95% CI for *IDH*2 mutations in MDS comparing with *IDH*2 wild-type by OS endpoints. (F) Forest plots of cumulative meta-analysis of *IDH* mutations in association with MDS for OS by published year.

The summary HRs for OS were 1.62 (95% CI, 1.27–2.09) for *IDH1/2* mutations (Figure 4A), and 2.21 (95% CI, 1.45–3.38) for *IDH1* mutations (Figure 4C), indicating that the presence of *IDH1* mutations was a negative prognostic factor for OS, whereas a marginal association was discovered for *IDH2* mutations 1.38 (95% CI, 0.95–2.02) (Figure 4E). Figure 4B and 4D showed the results of meta analysis for LFS, the summary HRs of LFS were 2.21 (95% CI, 1.48–3.30) for *IDH1/2* mutations and 2.65 (95% CI, 1.53–4.59) for *IDH1* mutations. There was moderate heterogeneity among studies (*I^2^*<75%), but no publication bias was found. Since significant heterogeneity across studies was detected, we executed sensitivity analyses and the results demonstrated the robust stability of the current results.

Cumulative analysis of the relationship between *IDH* mutations and MDS was performed via the assortment of studies by publication time. Inclinations toward significant association were evident over time. Moreover, the 95% CI became increasingly narrow with accumulation of more data, indicating the exactness of estimates was progressively boosted by the addition of more subjects (Figure 4F).

## Discussion

Due to the heterogeneity that still exists in the current prognostic scoring systems of MDS, the inclusion of novel molecular markers in these systems may enhance prognostic information. Although single gene mutations are not currently included in prognostic scoring systems, they may be vital to clinical phenotypes and overall survival in MDS. Actually, a great number of single gene mutations including *EZH2*, *SF3B1*, *TET2*, *ASXL1* and *TP53* have been associated with the development of MDS [25], [26]. The illumination of new gene mutations may therefore improve the prevention, diagnosis, prognosis and treatment of MDS.

IDH is a key cytosolic enzyme in the Krebs cycle. It catalyzes the decarboxylation of isocitrate to α-KG, leading to the production of nicotinamide adenine dinucleotide phosphate (NADP) [27], [28]. *IDH* mutations were first reported in a metastatic colon cancer in 2006 [29], and then since 2010 recurring *IDH* mutations were successively found in MDS (3.42%∼12.27%) [3], [14], [17], [30], [31], [32]. *IDH* mutations impair the normal enzymes’ function, which may be associated with poor prognosis in MDS. However, prior studies have not provided a definitive link between *IDH* mutations and MDS. Meta-analysis is a useful statistical method for integrating results from independent studies for a specified outcome. Combining the relevant studies increases statistical power and thus makes it possible to detect effects that may be missed by individual studies. Therefore, we summarized here the current data available regarding this potential relationship and revealed several valuable points.

Firstly we discovered a significant relationship between *IDH1/2* mutations status and MDS prognosis in the Chinese population, *IDH* mutations predicted more adverse OS for patients with MDS (*P* = 0.007). Furthermore, a meta-analysis combining the current and six previously published studies on *IDH1/2* mutations and MDS indicated *IDH1/2* mutations negatively affected OS (HR, 1.62; 95% CI, 1.27–2.09) and LFS 2.21 (95% CI, 1.48–3.30). Cumulative analysis further confirmed the significant correlation, demonstrating the effect of the variant became progressively significant with each accumulation of more data over time. In addition, when we conducted subgroup analyses, our data illustrated that *IDH1* but not *IDH2* mutations negatively affected OS 2.21 (95% CI, 1.45–3.38) and LFS 2.65 (95% CI, 1.53–4.59) in patients with MDS. Secondly, the presence of *IDH1/2* mutations might subdivide the intermediate-1 IPSS risk group as this was associated with a shorter OS in this group (*P* = 0.039). Finally, we found *IDH1/2* mutation-positive patients with MDS who were treated with decitibine had a significantly longer OS (*P* = 0.023) suggesting hypomethylating agents might be an effective treatment option for these patients.

There are several mechanisms by which *IDH1/2* mutations can worsen the prognosis of patients with MDS. (1) *IDH* mutations occur at low frequency (3.42%–12.27%) in MDS, but *IDH1/2* mutations are more frequent in both de novo AML (7.5%–31%) and AML arising from MDS (7.5%) [15], [33], [34], [35], [36], [37], indicating a role for *IDH* mutations in leukemic transformation of MDS. (2) At the cytogenetic level, Caramazza et al. [38] showed a likely association between *IDH1/2* mutations and trisomy 8 in MDS, and our results demonstrated 75% (3/4) of *IDH1/2* mutants with abnormal karyotypes carried a −7/7q- karyotype. In MDS, +8 and −7/7q- karyotypes were categorized in the intermediate-risk and high-risk cytogenetic group, respectively, suggesting they were linked to poor outcome in MDS. (3) The mutant IDH proteins displayed a gain of function as they could convert the α-KG that was generated by wild-type IDH proteins into 2-hydroxyglutarate (2-HG). Recent studies [39], [40] reported that 2-HG was closely related to therapeutic response and relapse in AML. Since MDS and AML share many similar characteristics [41], it is possible that 2-HG is an oncogenic factor in MDS. (4) DNA hypermethylation played a vital role in MDS pathogenesis [42]. Dang et al [43] reported that mutant IDH1/2 proteins produced 2-HG which competitively inhibited α-KG-dependent enzymes, such as the DNA demethylating protein TET2 (Ten-eleven translocation 2) resulting in DNA hypermethylation. Indeed, Figueroa et al. [44] found that AML patients with *IDH1/2* mutations shared a similar methylation profile to those with *TET2* mutations, and both mutations led to a block in myeloid differentiation and leukemogenesis. This might also be a potential reason for affecting outcomes in MDS. (5) Accumulation of 2-HG might lead to DNA damage by generating reactive oxygen species (ROS) [45] and inhibit EGLN (Egg-laying defective Nine) with subsequent stabilization of hypoxia-inducible factor 1α (HIF-1α) [46]. DNA damage and HIF-1α stabilization have been reported to be closely linked to MDS pathogenesis [47], [48]. There are thus several mechanisms by which *IDH1/2* mutations may contribute to MDS pathophysiology but further research is needed to elucidate their exact contributions to the disease.

While the findings of this study are largely consistent with previous studies on *IDH1/2* mutations in MDS, several limitations should be addressed. First, analyses were based on observational rather than experimental studies. Cohort studies are prone to several types of bias including selection bias and loss-to-follow-up [49]. Second, we did not uncover unpublished studies and chose to collect only published articles in English, which could bring publication bias, despite there being no significant evidence of publication bias observed in Egger’s test. Third, our study did not assess the potential effects of gene-gene interactions known to influence outcome in MDS such as *TET2* mutation-associated hypermethylation [44]. Similarly, we did not account for other known genetic contributions to leukemic transformation in MDS such as *ASXL1* loss-of-function [50]. This could lead to possible confounding in our study results. However, since *IDH1/2* mutations and *TET2* mutations were previously found to be mutually exclusive in patients with AML [44], it is not likely this particular interaction significantly contributed to our results.

In conclusion, we screened exon 4 of the *IDH1/2* gene in a large cohort of Chinese patients with MDS. Consistent with previous observations, we found that *IDH* mutations were present in some patients with MDS. *IDH1* mutations rather than *IDH2* mutations were significantly associated with shorter OS and LFS in patients with MDS. Further studies with larger sample sizes and functional assays of mutant IDH proteins are essential to decipher the role of *IDH* mutations in the development of MDS. Given that *IDH* mutations may adversely affect outcome in MDS are relatively easy to assess at diagnosis, examining *IDH* mutations in MDS may enhance the current prognostic scoring systems and guide patient-specific treatment in MDS. Finally, the identification of *IDH* mutations in the development and progression of MDS offers the promise of ameliorating the disease using targeted therapeutics against this biochemical pathway.

## Supporting Information

Checklist S1 PRISMA checklist.
(DOC)
